# *In Vitro* Anti-rotaviral Activity of *Achillea kellalensis*

**DOI:** 10.17795/jjnpp-8591

**Published:** 2013-07-17

**Authors:** Reza Taherkhani, Fatemeh Farshadpour, Manoochehr Makvandi

**Affiliations:** 1 Infectious and Tropical Diseases Research Center, Ahvaz Jundishapour University of Medical Sciences, Ahvaz, IR Iran; 2 Department of Medical Virology, School of Medicine, Ahvaz Jundishapur University of Medical Sciences, Ahvaz, IR Iran

**Keywords:** *Achillea*, Cell Survival, *In Vitro*

## Abstract

**Background:**

*Achillea kellalensis*, which is frequently used by Chaharmahal va Bakhtiarians residing in, Southwest of Iran, as a traditional herbal medicine for the treatment of acute diarrhea, has been selected to examine its antiviral activities against bovine rotavirus and cell toxicity activity in MA-104 cells.

**Objectives:**

The aim of this study was to evaluate the *in vitro* cytotoxic and anti-rotavirus properties of crude extracts of *A. kellalensis*.

**Materials and Methods:**

The dried and powdered flowers of *Achillea kellalensis* were extracted with hot water and ethanol 50% (v/v). The cell viability and toxicity of the extracts were evaluated on MA-104 cells using four methods; trypan blue dye, NR, crystal violet and MTT assay. The *in vitro* anti-rotavirus properties were determined via four different assays, in order to evaluate the direct inhibition and/or the inhibition of viral replication.

**Results:**

Cytotoxicity of two *A. kellalensis* extracts showed different concentrations. Hydro-alcoholic extract had low CC_50_ at 600 µg/mL by the NR assay while the aqueous extract had high CC_50_ at 1000µg/mL by the crystal violet method. In the simultaneous treatment assay and post treatment assay, the extracts were able to prevent viral replication and inhibit the viral CPE on MA-104 cells at 10 TCID_50_, but the extracts did not exhibit direct antiviral activity on rotavirus adsorption. The effective concentration (EC_50_) of both extracts was observed to be 100 µg/mL.

**Conclusions:**

These results indicate that *A. kellalensis* extracts exert potent anti-rotaviral activity only after viral adsorption. The two extracts from *A. kellalensis* showed a good selectivity index. Also these results suggest that extracts prepared from the flowers of *A. kellalensis* may be potential anti-rotaviral agents *in vivo* and be useful in veterinary medicine.

## 1. Background

Rotaviruses are the major etiologic agents that cause severe, acute dehydrating gastroenteritis in young children and in a wide variety of domestic animals. The virus is transmitted by the fecal-oral route. The clinical signs of rotavirus infection include acute gastroenteritis with fever, vomiting, abdominal pain, diarrhea, dehydration and rhinitis (1). Each year, rotavirus causes an estimated 2 million hospitalizations and 450,000 deaths in children worldwide (2). The only known treatment for rotavirus gastroenteritis is supportive therapy, the replacement of ﬂuids and electrolytes lost by vomiting and diarrhea (1). Oral immunoglobulins have also been used to treat diarrhea caused by rotaviruses; however, these are very costly with unknown side-effects (3). Two oral, attenuated, reassortant rotavirus vaccines (Rotarix and Rotateq) may prevent rotavirus infections in infants and young children (4), but they haven’t been approved for infants with immunosuppressive illnesses and also vaccine effectiveness in populations at the greatest risk in developing countries such as Asia and Africa remains to be assessed (5). Therefore, it is necessary to develop a new antiviral agent against this virus. Plants are rich in a broad range of secondary metabolites, such as flavonoids, polysaccharides, alkaloids, terpenoids, and tannins, phenolics and amino acids which have been found *in vitro* to have antimicrobial and antiviral properties (6).

Several antiviral plant extracts such as black tea, *Citrus aurantium*, marine sponges, soy isoﬂavones, *Stevia rebaudiana *and *Alpinia katsumadai* have been found to be inhibitory for rotaviruses *in vitro*, with less toxic, fewer side-effects and cheaper (7-13). However, these drugs are not currently available for human or animal use and none of them have yet been in clinical use. This has renewed interest in the search for a new anti-rotaviral agent. The genus Achillea belongs to Asteraceae or Compositae, the largest family of flowering and vascular plants (14). Achillea species have been used by local people as folk or traditional herbal medicines in various regions throughout the world. *Achillea kellalensis* *Boiss.* & *Hausskn*. originate from Chaharmahal va Bakhtiari, Southwest of Iran, is locally known as Golberrenjas or Bumadaran-e-Sabzekohi and the consumption of herbal teas prepared from flowers of this species has long been used as a medicinal plant especially for treatment of the acute diarrhea (15). Achillea species have major bioactive components, including flavonoids, terpenoids (monoterpenes, sesquiterpenes, diterpenes, triterpenes), camphor, borneol, lignans, amino acid derivatives, fatty acids and alkamides and the activity of this genus against different bacteria, fungi, viruses and parasites might be due to the presence of secondary active metabolites such as flavonoids, phenolic acids, terpenoids and sterols which have been isolated (16-18). However, to date the anti-rotavirus activities of aqueous and hydro-alcoholic extractions of *A. kellalensis* have not been evaluated. 

## 2. Objectives

In the present study we described *in vitro* cytotoxic and anti-RV properties of crude extracts of the *A. kellalensis* collected from Chaharmahal va Bakhtiari.

## 3. Materials and Methods

### 3.1. Preparation of Aqueous and Hydro-alcoholic *A. kellalensis* Extracts

*A. kellalensis* flowers were cleaned, air-dried and ground to a fine texture with a grinder. For aqueous extract (hot water extract), 200 mL of boiling distilled water was added to 10 g of powdered flowers in an Erlenmeyer flask and kept in a shaker at 90 rpm for 1 h at room temperature. The brewed extract was filtered through gauze and clarified by centrifugation at 3000 rpm for 10 minutes; the supernatant was then dried in an electric water bath at 50˚C. Hydro-alcoholic extraction was performed by soaking 10 g of powdered flowers in 200 mL of 50% ethanol and kept in a shaker at 90 rpm for 2 days at room temperature (20˚C) and then filtered through gauze. The extractions were filtered through a Whatman No 1 filter paper, then the filtrate evaporated to dryness in an electric water bath at 50˚C. 1 g of each of the dried extracts were suspended in 5 mL of respective solvents and sterilized by ﬁltration through a 0.22 µm ﬁlter (Jet Biofil), stored at 4˚C.

### 3.2. Cell Culture

MA-104 cells (Embryonic rhesus monkey kidney cells) were cultured as a monolayer in DMEM supplemented with 10% fetal bovine serum (FBS) and 2 mM L-glutamine in the presence of penicillin G (100 U/mL), streptomycin (100 µg/mL), gentamycin (50 µg/mL) and amphotericin B (2.5 µg/mL) at 37˚C in a humidiﬁed atmosphere containing 5% CO_2_ / 95% air.

### 3.3. Cell Toxicity and Viability (Determination of the Maximum Non-toxic Concentration)

Cytotoxicity was assessed by microscopic observation, neutral red assay, crystal violet assay and MTT reduction assay and viability of cells was determined by the trypan blue dye exclusion method. MA-104 cells were seeded at a density of 1 × 10^4^ cells per well (in 200 µL medium) of 96-well plates (Nunc) by an 8-channel pipette and 5 × 10^4^ cells per well (in 1 mL medium) of 24-well culture plates. After 48 h of incubation at 37˚C in 5% CO_2_ atmosphere, the growth medium was replaced by similar volumes of fresh medium free of FBS with a series of different concentrations (15 doses, maximum 2 mg/mL and minimum 0.05 mg/mL) of each herbal extract while positive (the cells were incubated with 1 mM hydrogen peroxide) and negative controls (untreated cells that received only the medium without any extraction) were prepared. All the treated cells were re-incubated at 37˚C for 72 h for different cytotoxic assays. The highest concentration of the extract showing no cell toxicity was considered as the maximum non-toxic concentration (MNTC). The 50% cytotoxic concentration (CC_50_) was estimated by regression analysis.

#### 3.3.1. MTT Assay 

The MTT cytotoxicity assay was carried out with slight modiﬁcations as previously described by Mosmann (19). The percentage of cytotoxicity was calculated as (A-B)/A × 100, where A is the mean optical density of untreated wells and B is the mean optical density of wells with plant extracts. For each extract, the obtained CC_50_ value was deﬁned as the concentration that reduced the absorbance of treated cells by 50% when compared to untreated cells and this was determined by regression analysis (19).

#### 3.3.2. Neutral Red Dye Retention Assay

The NR assay is a cheap and sensitive method for the cytotoxicity test. The procedure was performed using a miner modification method as previously described. Plates were prepared similarl to that described for the MTT reduction assay (20).

#### 3.3.3. Crystal Violet Assay

At the end of the treatment period of 96-well microplates (72 h), with different concentrations of each extract in 4 replicates, the medium was removed and the trays were stained with a mixture of formalin 10% and crystal violet 0.13% in PBS for 10-20 minute. The plates were rinsed twice with water and dried by inversion on filter paper and were then enumerated by measuring absorbance at 492 nm on an ELISA reader (Bio Rad). Reported values are the mean of 4 replicates. The percentage of cytotoxicity was calculated similar to that described for the MTT reduction assay (21).

#### 3.3.4. Trypan Blue Dye Uptake to Assess Viability

To determine the number of viable cells, the trypan blue exclusion method was performed. At the end of the treatment period of 24-well plates (72 h), with a series of various concentrations of each extract in 4 replicates (0.5 mL/well), the medium was removed and the cells were detached from the wells by trypsin-EDTA solution. The solution, including the detached cells, was centrifuged at 1000 rpm for 10 min and the supernatant was removed. The detached cells in each well were stained with 0.4% trypan blue dye, transferred to the hematocytometer and were counted using an optical microscope (Olympus). Cell viability percentage was calculated by the division of the total counted number of viable cells by total cells (100% viability). For minimal viability, the cells were incubated with 1 mM hydrogen peroxide, which resulted in total cell death evident by trypan blue viability staining (positive control). The untreated cells that received only the medium without any extraction were used for maximum viability (negative control). The reported values are the mean of 4 replicates (22).

### 3.4. Titration of the Virus

Virus titration on conﬂuent monolayers of MA-104 cells was performed by the limit-dilution method. Ten-fold serial dilutions of the virus stock were made up (10-1 to 10-10) in DMEM free of FBS, with 10 µg/mL of trypsin (TPCK treated). The titer of propagated viral stock was determined by using the MTT colorimetric technique, crystal violet assay and CPE observation of MA-104 cells and was expressed as 50% tissue culture infectious dose (TCID_50_) per mL by the Reed-Munch method. The determined titer was 106 TCID_50_/mL (23).

### 3.5. Antiviral Assays

The antiviral assays used in this study have been previously described (11, 24, 25) and the visualization of these assays was also performed by the same methods used to evaluate cell toxicity and MTT reduction assay, crystal violet method, neutral red assay and CPE observation as described above were followed. Microscopic examination for CPE was performed with scores being from 0 (normal cells) to 4 (100% CPE). The 50% effective concentration (EC_50_) was deﬁned as the reciprocal of concentration of each extract required to prevent rotavirus-induced cytolysis by 50%.

#### 3.5.1. Virucidal Assay

Virucidal assay (Adsorption Inhibition Assay)‪‪‪‪‪ was performed to determine the ability of the extracts to inhibit or decrease the rate of adsorption or penetration of the rotavirus on the treated MA-104 cells. MA-104 cells were seeded at the density of 1 × 10^4^ cells per well (in 200 µL medium) onto 96-well plates (Nunc) and incubated at 37˚C in 5% CO_2_ atmosphere for 48 h. In the mixed treatment assay (virucidal assay), 10 TCID_50_ of activated rotaviruses were mixed with various non-toxic concentrations (0.05–0.5 mg/mL) of each extract and incubated at 4˚C for 1 h. The mixtures were added to each well (0.2 mL/well) and incubated at 37˚C for 1 h. The solution was removed. The cells were washed with PBS, to remove the remaining extract from the wells, and the wells were replenished with DMEM containing 1 µg/mL trypsin (0.2 mL/well). The cells were incubated for 72 h at 37˚C under 5% CO_2_ atmosphere. Experiments were carried out in 4 replicates. The 50% effective concentration (EC_50_) was estimated by regression analysis (24).‬‬‬‬‬‬‬‬‬‬‬‬‬‬‬‬‬‬‬‬

#### 3.5.2. Determination of Antiviral Activity

CPE inhibition assay was performed to determine whether the extracts prevented viral replication by affecting one or more steps of their replication when viruses were already in the cells.

A) In the simultaneous treatment assay (11), MA-104 cells were seeded as previously described. After the incubation period, the spent medium was decanted and the cells were then washed two times using PBS, and medium containing 10 TCID_50_ of activated rotaviruses with different non-toxic concentrations (maximum 0.5 mg/mL and minimum 0.05 mg/mL) of each crude extract were added to each well (0.2 mL/well). The plates were incubated at 37˚C for 72 h in a humidiﬁed incubator with a 5% CO_2_/95% air atmosphere. Experiments were carried out in 4 replicates. The 50% effective concentration (EC_50_) was estimated by regression analysis.

B) In the post treatment assay (viral replication inhibition assay) (25), MA-104 cells were seeded as a previously described. After the incubation period, the growth medium was removed and the cells were washed two times using PBS and then 10 TCID_50_ of activated rotaviruses were inoculated onto each well (50 µL/well) for 2 h. The inoculum was removed, cell monolayers were washed one time using PBS and replaced by DMEM containing 1 µg/mL trypsin and various non-toxic concentrations (0.05–0.5 mg/mL) of each extract (0.2 mL/well). The cells were incubated for 72 h at 37˚C under 5% CO_2_ atmosphere. Experiments were carried out in 4 replicates. The 50% effective concentration (EC_50_) was estimated by regression analysis. 

C) In the pretreatment assay (11), after a 48 h period of incubation, various non-toxic concentrations (0.05–0.5 mg/mL) of each extract (0.2 mL/well) were added to the cells and incubated for 15 h prior to virus infection (10 TCID_50_/well). The cells were incubated for 72 h at 37˚C under 5% CO_2_ atmosphere. Experiments were carried out in 4 replicates. The 50% effective concentration (EC_50_) was estimated by regression analysis.

Controls consisting of virus control wells or untreated-infected cells (with virus but without any extracts), toxicity control wells or treated non-infected cells (with various concentrations of each crude extract but without virus), cell control wells or untreated non-infected cells (that received only the medium containing 1 µg/mL of trypsin without any extraction and virus), were used simultaneously. 

### 3.6. Data Analysis

The CC_50_ and EC_50_ for each extract were calculated from concentration-effect curves after regression analysis. The therapeutic index (TI) or selective index (SI) for each extract is defined as the ratio between the maximum drug concentration at which 50% of the growth of normal cells is inhibited (CC_50_), and the minimum drug concentration at which 50% of the virus is inhibited (EC_50_) (SI = CC_50_/EC_50_) (26).

## 4. Results

**Figure 1.**
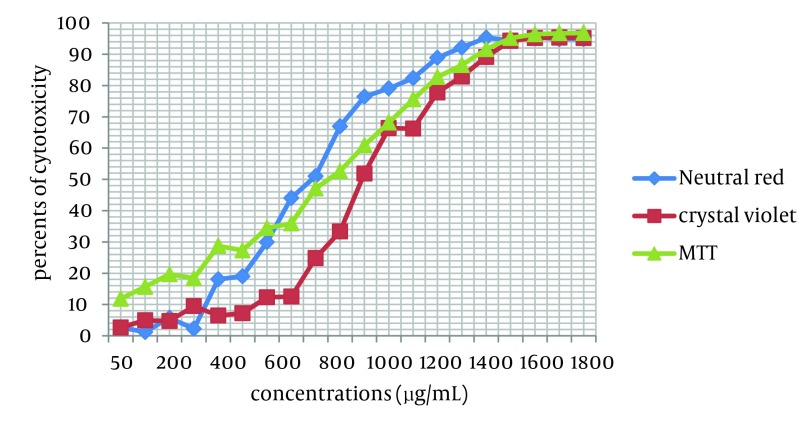
Cytotoxicity in MA-104 Cells Induced by Exposure to Different Concentrations of Aqueous *A. kellalensis* Extract and Assessed by Crystal Violet Method, NR and MTT Assays

**Figure 2.**
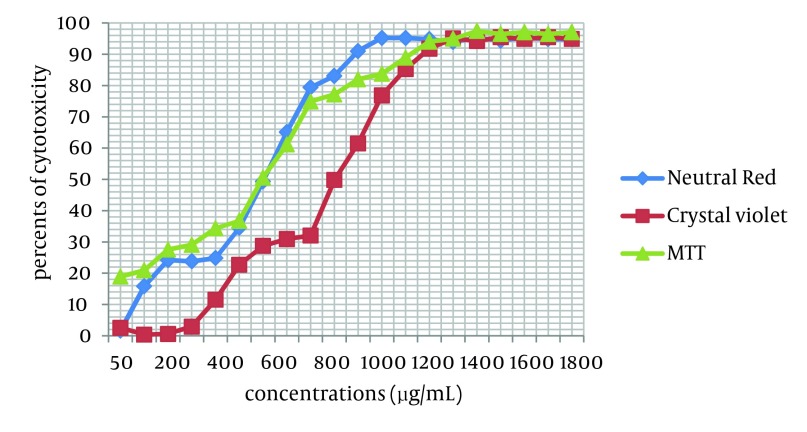
Cytotoxicity in MA-104 Cells Induced by Exposure to Different Concentrations of Hydro-alcoholic *A. kellalensis* Extract and Assessed by Crystal Violet Method, NR and MTT Assays

Cytotoxicity of two *Achillea kellalensis *extracts showed different concentrations. Hydro-alcoholic extract had low CC_50_ at 600 µg/mL by the NR assay while aqueous extract had high CC_50_ at 1000 µg/mL by the crystal violet method (Table 1, Figures 1 and 2). 

**Table 1.** Inhibitory and Cytotoxicity Effects of *A. kellalensis *Extracts Against Rotavirus in the Simultaneous and Post Treatment Assay ^a ^

Methods	Aqueous Extract			Hydro-alcoholic Extract		
	CC_50_, µg/mL	EC_50_, µg/mL	SI	CC_50_, µg/mL	EC_50_, µg/mL	SI
**Neutral Red**	700	100	7	600	100	6
**Crystal Violet**	1000	100	10	900	100	9
**MTT**	800	100	8	700	100	7

^a^ CC_50_: Concentration of Extract that is Cytotoxic to 50% of cells; EC_50_: Concentration of Extract That Inhibits Viral Infectivity (Cytopathic Effect) by 50%; SI: selectivity index = CC_50_/EC_50_ = the Mean Values of 4 Replicate Experiments are Shown.

**Figure 3.**
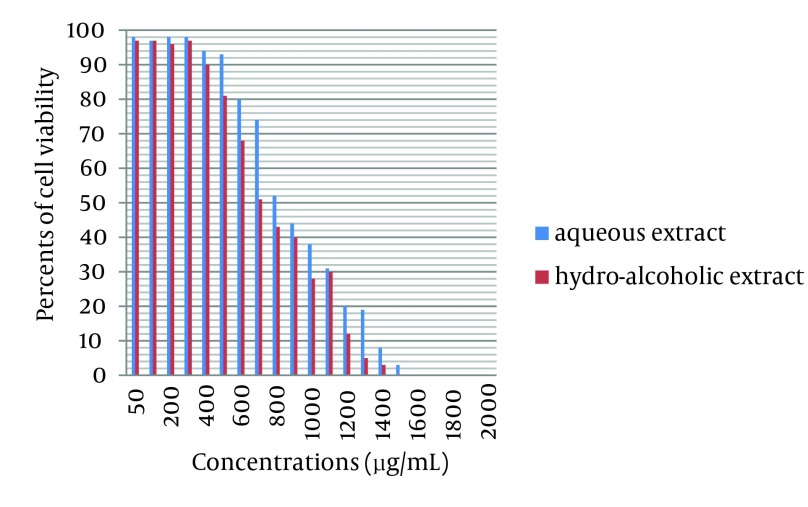
Effect of Different Concentrations of Aqueous and Hydro-alcoholic Extracts of *A. kellalensis* on Cell Viability as Measured by Trypan Blue Dye Exclusion Method Compared with positive control (0 µg/mL) with 98% cell viability for aqueous extract and 97% cell viability for hydro-alcoholic extract

Growing MA-104 cells were treated with various concentrations of *Achillea kellalensis *extracts and the viability was measured by trypan blue dye exclusion method. In MA-104 cells a decrease in the viability with increasing concentration was observed. The aqueous and hydro-alcoholic extracts showed 50% viability at 800 µg/mL and 700 µg/mL respectively. These data are shown in Figure 3. The inhibitory effects of *A. kellalensis *on the bovine rotavirus were examined by the same methods used to evaluate cell toxicity. Extracts from flowers of *Achillea kellalensis *were shown to be effective against bovine rotavirus. These results of the antiviral assays of *A. kellalensis *extracts are depicted in Table 1. 

In the simultaneous treatment assay and post treatment assay, the extracts were able to prevent viral replication and inhibit the viral CPE on MA-104 cells at 10 TCID_50_. The effective concentration (EC_50_) of both extracts was observed at 100 µg/mL. In order to test the ability of the two *A. kellalensis* extracts in inhibiting the adsorption of bovine rotaviruses in MA-104 cells, the virucidal assay was used. However, in contrast to the simultaneous treatment assay and post treatment assay, no inhibitory effects against rotavirus was shown in the virucidal assays and pretreatment assay, and the extracts did not exhibit direct antiviral activity on rotavirus adsorption.

## 5. Discussion

The present results support the ethnomedical use of *A. kellalensis* as anti-rotavirus agent. This is the first report on the anti-rotaviral and cytotoxic activity of *A. kellalensis*. Our results indicate that of the 3 methods examined to determine cell toxicity of extracts, neutral red assay was the most sensitive. Time-of-addition experiments were carried out to evaluate the step at which *A. kellalensis* extracts exert inhibitory activities. The extracts were able to inhibit viral replication and prevented the formation of CPE in infected cells.

In the present study the simultaneous treatment assay results are in agreement with the post treatment assay results, indicating that the *A. kellalensis* extracts could probably exert a potential anti-rotavirus activity via blockage of RNA synthesis. However, in the virucidal assay, the extracts were not able to inhibit or decrease the rate of adsorption or penetration of the rotavirus in the treated MA-104 cells. The extracts were not able to inhibit viral replication when the amount of virus increased from 10 to 100 TCID_50_ when they were examined at a concentration of 500 µg/mL. These results indicate that *A. kellalensis* extracts exert potent anti-rotaviral activity only after viral adsorption. The two extracts from *A. kellalensis* showed a good selectivity index. *A. kellalensis* is a traditional medicinal herb and has long been used to treat acute gastritis (15). Wound healing activity (27), antihypertensive and antihyperlipidemic effects (28), antioxidant activity (29), estrogenic activity (30), anti-diabetic activity (31), antispermatogenic effect (32), antiulcer activity (33), anti-bacterial and anti-fungal activity (17, 34, 35), anti-epimastigote activity (36), anti-viral activity (37), antispasmodic activity (38) and anti-inflammatory activity (39), of various species of Achillea have been reported. In an earlier investigation where the *Achillea fragrantissima* extracts had been examined for *in vitro* anti-poliovirus activity, a significant antiviral activity was also observed (37). The activity of *A. kellalensis* extracts against bovine rota virus might be due to the presence of secondary active metabolites such as flavonoids (9) and phenolic (16-18, 24) acids which have been shown to possess blocking RNA synthesis activity (40). At this point, our data demonstrated that *A. kellalensis* extracts have potent anti-rotavirus effects *in vitro*. However, further investigations are required to explore anti-rotavirus activities *in vivo* with bovine rotavirus strains in calves or murine rotavirus strains in mice. Considering that rotaviruses cause severe diarrhea and mortality in calves and economic losses in the dairy industry, therefore use of this herb can be useful in veterinary medicine.

## References

[1] Estes MK, Kapikian AZ, Knipe DM, Grifﬁn DE, Lamb RA, Straus SE, Howley PM, Martin MA (2007). Rotaviruses.. Fields Virology..

[2] Tate JE, Burton AH, Boschi-Pinto C, Steele AD, Duque J, Parashar UD (2012). 2008 estimate of worldwide rotavirus-associated mortality in children younger than 5 years before the introduction of universal rotavirus vaccination programmes: a systematic review and meta-analysis.. Lancet Infect Dis..

[3] Guarino A, Canani RB, Russo S, Albano F, Canani MB, Ruggeri FM (1994). Oral immunoglobulins for treatment of acute rotaviral gastroenteritis.. Pediatrics..

[4] Ruiz-Palacios GM, Perez-Schael I, Velazquez FR, Abate H, Breuer T, Clemens SC (2006). Safety and efficacy of an attenuated vaccine against severe rotavirus gastroenteritis.. N Engl J Med..

[5] Dennehy PH (2008). Rotavirus vaccines: an overview.. Clin Microbiol Rev..

[6] Cowan MM (1999). Plant products as antimicrobial agents.. Clin Microbiol Rev..

[7] Andres A, Donovan SM, Kuhlenschmidt TB, Kuhlenschmidt MS (2007). Isoflavones at concentrations present in soy infant formula inhibit rotavirus infection in vitro.. J Nutr..

[8] Andres A, Donovan SM, Kuhlenschmidt MS (2009). Soy isoflavones and virus infections.. J Nutr Biochem..

[9] Bae EA, Han MJ, Lee M, Kim DH (2000). In vitro inhibitory effect of some flavonoids on rotavirus infectivity.. Biol Pharm Bull..

[10] Clark KJ, Grant PG, Sarr AB, Belakere JR, Swaggerty CL, Phillips TD (1998). An in vitro study of theaflavins extracted from black tea to neutralize bovine rotavirus and bovine coronavirus infections.. Vet Microbiol..

[11] da Silva AC, Kratz JM, Farias FM, Henriques AT, Dos Santos J, Leonel RM (2006). In vitro antiviral activity of marine sponges collected off Brazilian coast.. Biol Pharm Bull..

[12] Takahashi K, Matsuda M, Ohashi K, Taniguchi K, Nakagomi O, Abe Y (2001). Analysis of anti-rotavirus activity of extract from Stevia rebaudiana.. Antiviral Res..

[13] Kim HH, Kwon HJ, Ryu YB, Chang JS, Cho KO, Hosmillo MD (2012). Antiviral activity of Alpinia katsumadai extracts against rotaviruses.. Res Vet Sci..

[14] Mozaffarian V (1996). A Dictionary of Iranian Plant Name.

[15] Ghasemi PA (2009‏‎). Medicinal plants used in Chaharmahal and Bakhtyari‎districts, Iran..

[16] Si XT, Zhang ML, Shi QW, Kiyota H (2006). Chemical constituents of the plants in the genus Achillea.. Chem Biodivers..

[17] Aburjai T, Hudaib M (2006). Antiplatelet, antibacterial and antifungal activities of Achillea falcata extracts and evaluation of volatile oil composition.. Pharmacog Mag..

[18] Rustaiyan A, Masoudi S, Yari M (1999). The essential oils of Achillea aucheri Boiss. and A. Kellalensis Boiss. et Hausskn. from Iran.. J Essent Oil Res..

[19] Mosmann T (1983). Rapid colorimetric assay for cellular growth and survival: application to proliferation and cytotoxicity assays.. J Immunol Methods..

[20] Repetto G, del Peso A, Zurita JL (2008). Neutral red uptake assay for the estimation of cell viability/cytotoxicity.. Nat Protoc..

[21] Flick DA, Gifford GE (1984). Comparison of in vitro cell cytotoxic assays for tumor necrosis factor.. J Immunol Methods..

[22] Oh S, Daraio C, Chen LH, Pisanic TR, Finones RR, Jin S (2006). Significantly accelerated osteoblast cell growth on aligned TiO2 nanotubes.. J Biomed Mater Res A..

[23] Reed L Jj, Muench Hugo (1938). A simple method of estimating fifty per cent endpoints.. Am J Epidemiol..

[24] Kwon HJ, Kim HH, Ryu YB, Kim JH, Jeong HJ, Lee SW (2010). In vitro anti-rotavirus activity of polyphenol compounds isolated from the roots of Glycyrrhiza uralensis.. Bioorg Med Chem..

[25] Barnard DL, Hill CL, Gage T, Matheson JE, Huffman JH, Sidwell RW (1997). Potent inhibition of respiratory syncytial virus by polyoxometalates of several structural classes.. Antiviral Res..

[26] De Meyer N, Haemers A, Mishra L, Pandey HK, Pieters LA, Vanden Berghe DA (1991). 4'-Hydroxy-3-methoxyflavones with potent antipicornavirus activity.. J Med Chem..

[27] Pirbaloutl AG (2009). Medicinal plants used in Chaharmahal and Bakhtyari districts of Iran.. Herba Polonica..

[28] Asgary S, Naderi GH, Sarrafzadegan N, Mohammadifard N, Mostafavi S, Vakili R (2000). Antihypertensive and antihyperlipidemic effects of Achillea wilhelmsii.. Drugs Exp Clin Res..

[29] Konyalioglu S, Karamenderes C (2005). The protective effects of Achillea L. species native in Turkey against H(2)O(2)-induced oxidative damage in human erythrocytes and leucocytes.. J Ethnopharmacol..

[30] Innocenti G, Vegeto E, Dall'Acqua S, Ciana P, Giorgetti M, Agradi E (2007). In vitro estrogenic activity of Achillea millefolium L.. Phytomedicine..

[31] Yazdanparast R, Ardestani A, Jamshidi S (2007). Experimental diabetes treated with Achillea santolina: effect on pancreatic oxidative parameters.. J Ethnopharmacol..

[32] Montanari T, de Carvalho JE, Dolder H (1998). Antispermatogenic effect of Achillea millefolium L. in mice.. Contraception..

[33] Potrich FB, Allemand A, da Silva LM, Dos Santos AC, Baggio CH, Freitas CS (2010). Antiulcerogenic activity of hydroalcoholic extract of Achillea millefolium L.: involvement of the antioxidant system.. J Ethnopharmacol..

[34] Kordali S, Cakir A, Akcin TA, Mete E, Akcin A, Aydin T (2009). Antifungal and herbicidal properties of essential oils and n-hexane extracts of Achillea gypsicola Hub-Mor. and Achillea biebersteinii Afan.(Asteraceae).. Industr Crop Prod..

[35] Stojanovic G, Radulovic N, Hashimoto T, Palic R (2005). In vitro antimicrobial activity of extracts of four Achillea species: the composition of Achillea clavennae L. (Asteraceae) extract.. J Ethnopharmacol..

[36] Gohari AR, Saeidnia S, Hadjiakhoondi A, Naghinejad A, Yagura T (2008). Trypanocidal activity of some medicinal plants against the epimastigotes of Trypanosoma cruzi.. J Med Plant..

[37] Soltan MM, Zaki AK (2009). Antiviral screening of forty-two Egyptian medicinal plants.. J Ethnopharmacol..

[38] Karamenderes C, Apaydin S (2003). Antispasmodic effect of Achillea nobilis L. subsp. sipylea (O. Schwarz) Bassler on the rat isolated duodenum.. J Ethnopharmacol..

[39] Hegazy MEF, Abdel-Lateff A, Gamal-Eldeen AM, Turky F, Hirata T, Pare PW (2008). Anti-inflammatory activity of new guaiane acid derivatives from Achillea coarctata.. Nat Prod Comm..

[40] Jassim SA, Naji MA (2003). Novel antiviral agents: a medicinal plant perspective.. J Appl Microbiol..

